# Supplementary material for: Nonlinear mechanics of lamin filaments and the meshwork topology build an emergent nuclear lamina
Source: Nat Commun. 2020 Dec 4;11:6205. doi: 10.1038/s41467-020-20049-8 (PMC7718915; doi:10.1038/s41467-020-20049-8)
Supplement: Supplementary file 1 — Supplementary Information [file 41467_2020_20049_MOESM1_ESM.pdf]

Supplementary Data

Nonlinear mechanics of lamin filaments and the meshwork topology build an emergent nuclear lamina

K. Tanuj Sapra[1,2*], Zhao Qin[3], Anna Dubrovsky-Gaupp[1], Ueli Aebi[4], Daniel J. Müller[2], Markus J. Buehler[3], Ohad Medalia[1*]

[1] Department of Biochemistry, University of Zurich, Winterthurerstrasse 190, 8057 Zurich, Switzerland

[2] Department of Biosystems Science and Bioengineering, ETH Zurich, Mattenstrasse 26, 4058 Basel, Switzerland

[3] Laboratory for Atomistic and Molecular Mechanics,, Massachusetts Institute of Technology, Cambridge, Massachusetts 02139, USA

[4] Biozentrum, University of Basel, CH-4056 Basel, Switzerland

[#] Present address: Department of Civil and Environmental Engineering, Syracuse University, Syracuse, NY 13244, USA

Contents

Note 1

Figures 1 – 21

Tables 1 and 3

Movies 1 and 2

Note 1

Lamin filament mechanics under force

We first consider the deformation mechanics in the reversible low force regime assigned to the unfolding of the α-helical coiled-coil domain[1]. A lamin filament of length, $l \approx 54$ nm (considering only the rod domain, 352 amino acids), loaded in the center normal to the filament axis undergoes an initial deformation, $d_{low} \approx 54$ nm. At an average plateau force, $F_{low} \approx 0.3$ nN, the filament bends $\theta \approx 63^o$ with respect to the long axis of the filament [$\theta = \tan^{-1}(d_{low} / (0.5 \times l))$]. This is in agreement with a nuclear membrane deformation of $\theta \approx 58^o$ in cells spread on nano-pillars of radius 100 nm[2].

Thus, the lamin filament (radius, $r \approx 2$ nm) under an engineering stress, $\sigma_{plateau} \approx 24$ MPa, is stretched to a length (L_{low}) ≈61 nm (122 nm the entire length) [$L = d_{low} / \sin(\theta)$]. The engineering strain, ε_{low}, in the low force regime is estimated to be ≈126% similar to that measured for a vimentin dimer by atomistic simulations[3]. Because the plateau is reversible, we estimate the Young's modulus to be ≈19 MPa.

Next, we estimated the mechanical parameters in the high force regime of the FE curve where the filament stiffens. Similar to the plateau region, the loading is at the center and normal to the filament axis. At failure load, $F_{high} \approx 3$ nN, the filament was deformed to $d_{high} \approx 91$ nm. At yield stress, $\sigma_{yield} \approx 239$ MPa, the filament bends $\theta \approx 73^o$ relative to its long axis. Stretched to a length (L_{high}) of ≈95 nm (190 nm the entire filament), the engineering strain (ε_{high}) of the filament is ≈250%. In such a scenario, the force acting along the filament axis (F_A) is ≈3.1 nN, similar to $F_{high} \approx 3$ nN (normal to the axis). Thus, the bond loading in a filament is presumably independent of the directionality of the force acting on the filament, *i.e.*, whether the filament is bent or stretched. This phenomenon is also illustrated in the similarity of FE curves obtained by pushing (**Fig. 1, Supplementary Fig. 6**) or stretching (**Supplementary Fig. 7**)[1] lamin filaments in a meshwork.

Model of mechanical bending of a lamin filament

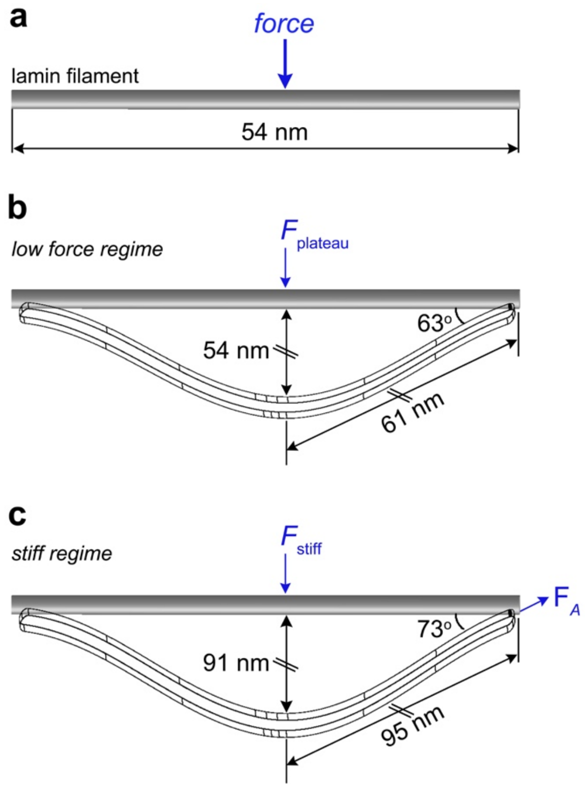

(**a**) Applying a force in the center of a 54 nm lamin filament results in a transformation through a reversible low force regime (**b**), followed by stiffening associated with α-helix to β-sheet transition (**c**).

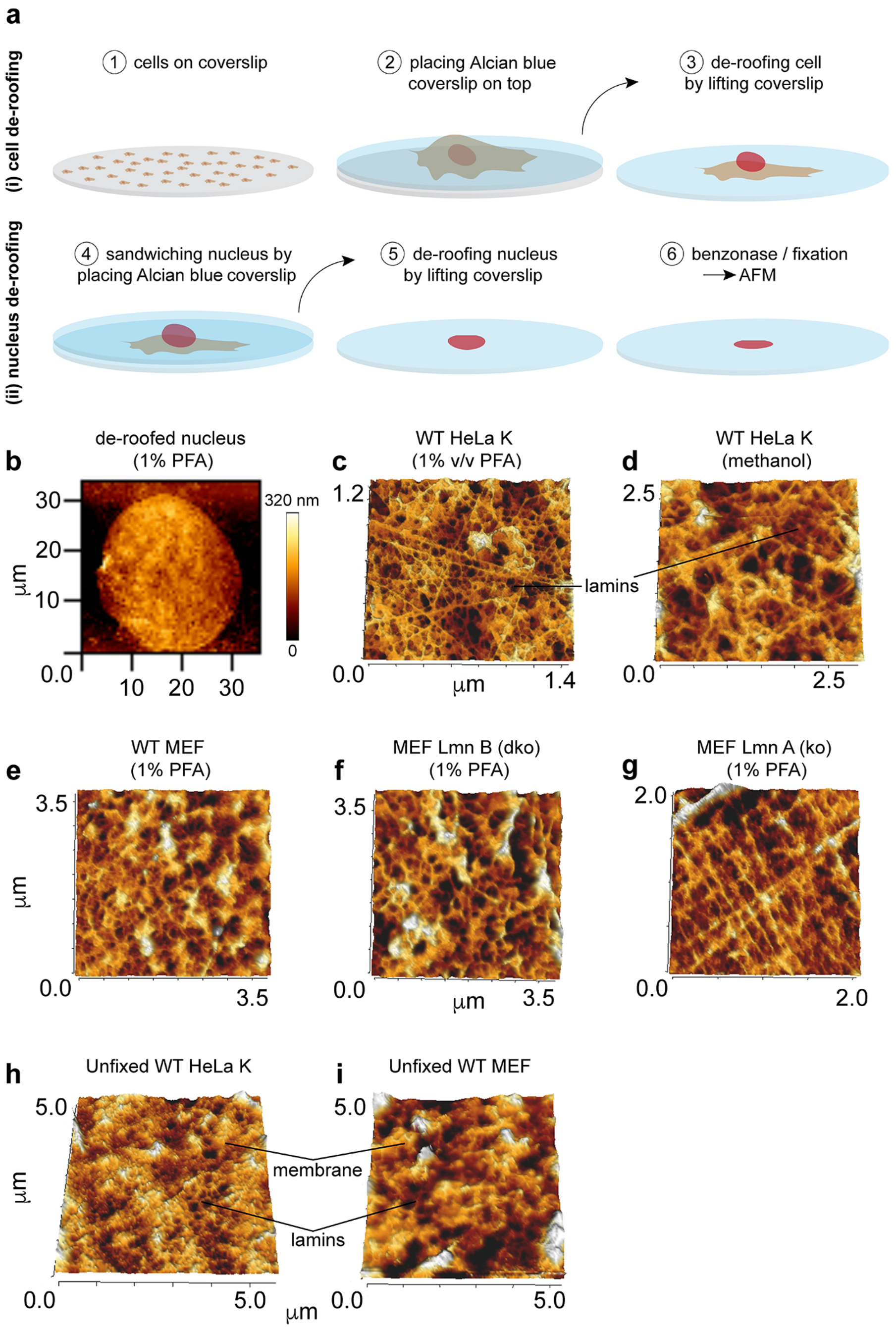

Supplementary Fig. 1. Imaging of mammalian nuclear lamina by AFM. (**a**) A schematic description of the procedure used for imaging mammalian nuclear lamina with AFM. HeLa cells seeded on a coverslip were de-roofed by placing and lifting an Alcian blue-coated coverslip. This facilitated the transfer of de-roofed cells with intact nuclei onto the Alcian blue coverslip. Nuclei were de-roofed in a second step by again placing and lifting an Alcian blue-coated coverslip. The open nuclei lacking NPCs were chemically fixed and imaged with AFM showing filamentous structures. (**b**) A typical AFM topograph of a de-roofed nucleus obtained by quantitative imaging. Color scale denotes the height of the topograph. (**c, d**) Images of the lamin meshwork from wild-type (WT) HeLa K cells, chemically fixed with 1% v/v PFA and methanol, respectively ($n \geq 5$ nuclei). (**e – g**) Images of lamin meshwork from wild-type, B-type lamin knockout and A-type lamin knockout mouse embryonic fibroblasts (MEF) fixed with 1% v/v PFA[4].

Most likely the NPCs were removed during the de-roofing and therefore were not observed ($n \geq 5$ nuclei). (**h, i**) Nuclei that were not chemically fixed did not show a clear meshwork as observed in fixed nuclei ($n \geq 5$ nuclei). Therefore, mechanical measurements on single lamin filaments were not performed on unfixed mammalian nuclei.

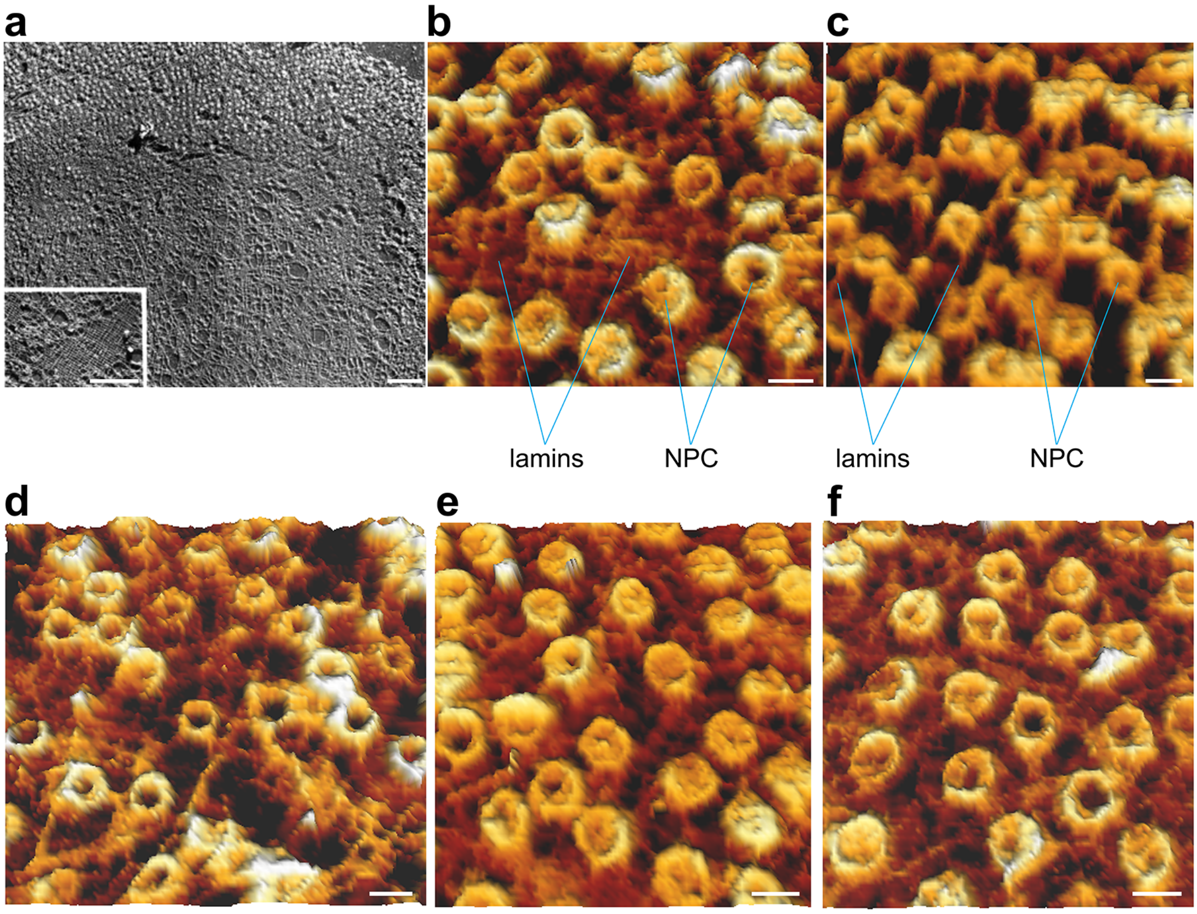

Supplementary Fig. 2. Electron microscopy and atomic force microscopy imaging of *X. laevis* oocyte nuclear envelopes (NEs). (**a**) Previously published image of a NE showing lamin meshwork and NPCs. The image was obtained using detergent treated, freeze-dried / metal-shadowed NE from *X. laevis* oocyte nucleus[5]. The meshwork shows lamin filaments arranged orthogonally and in an apparently random manner. Image used with permission from ref. *5*. Scale bars, 1000 nm. (**b – f**) Images of NEs obtained in this study from *X. laevis* oocyte nuclei that were isolated and manually opened to expose the nucleoplasmic face of the organelle. The orthogonal arrangement of the lamin filaments in the meshwork (and the NPCs) was clearly resolved by quantitative force imaging with AFM. Other regions of the samples showed less ordered lamin filament organization at the NE. Mechanical measurements on lamin filaments were performed in both meshwork types (N = 51, N is the number of independent experiments). Scale bars, 100 nm.

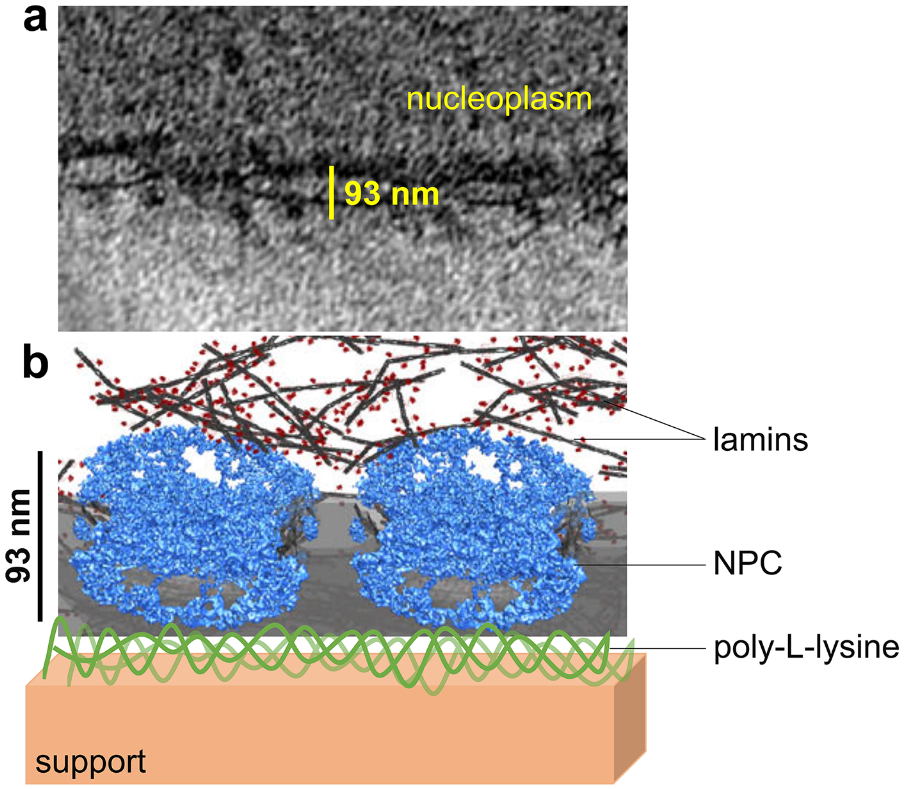

Supplementary Fig. 3. Lamin meshwork is situated at a distance of >93 nm from the surface support. (**a**) An *x-y* slice through a tomogram showing the distance between the INM and the cytoplasmic densities at the ONM, at the NE of a *X. laevis* oocyte (adapted from ref. 6). (**b**) A schematic view of our experimental setup. The glass surface was coated with poly-L-lysine. On top of the coated surface, the nuclear envelope was spread. Therefore, the lamin filaments and the glass support were separated by ≈100 nm.

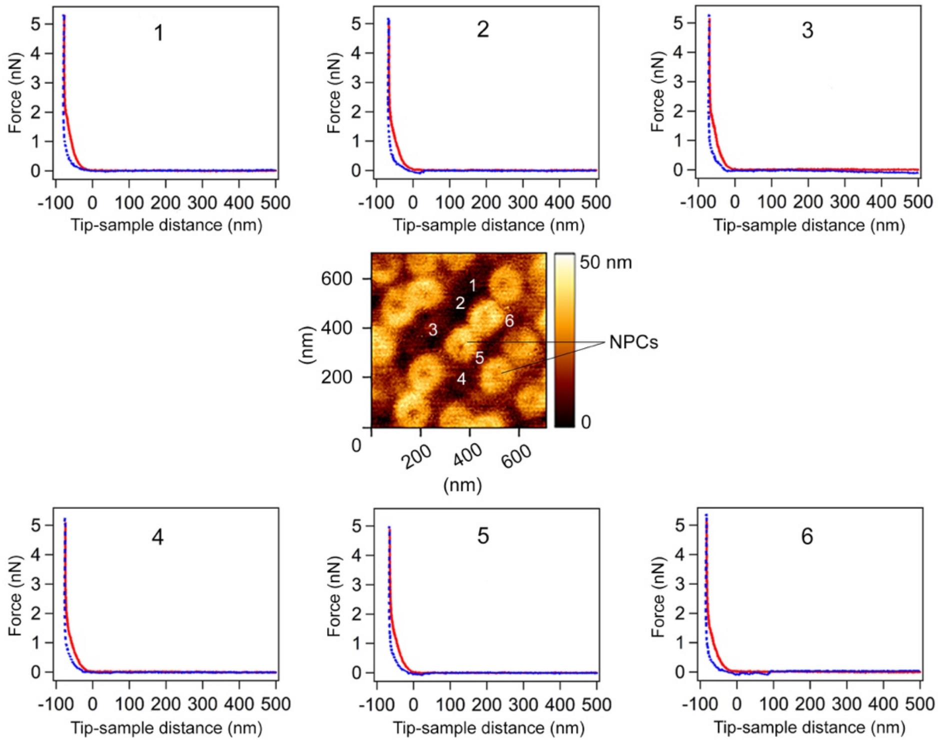

Supplementary Fig. 4. FE curves on nuclear membrane do not show force peaks. An AFM topograph was recorded by FE curve imaging. The region showed only NPCs but no lamin filaments. Positions marked 1-6 were chosen in between the NPCs, and the cantilever tip pushed in the active feedback mode. No distinct force peaks were observed in the FE curves as observed in the curves obtained by pushing lamin filaments (**Supplementary Figs. 5, 10**). Red curves denote the approach (pushing) of the cantilever tip, blue curves denote the retract. The numbers (1-6) on the AFM topograph correspond to the numbers labelling the FE curves. Color scale denotes the height of the AFM topograph.

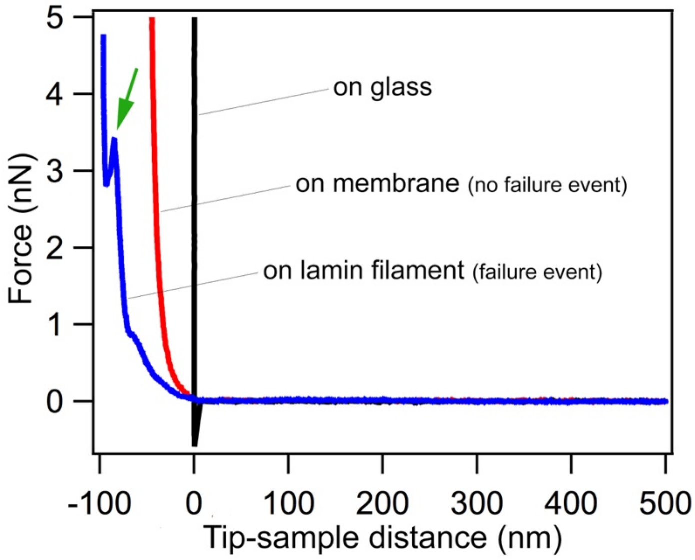

Supplementary Fig. 5. FE curves on glass, on membrane and on lamin filament show different profiles. The blue FE curve obtained by pushing a lamin filament shows an apparent failure event between 3 nN and 4 nN. On nuclear membrane (red FE curve) and bare glass without poly-L-lysine coating (black FE curve), no force peaks were detected. The FE curves clearly show that the surfaces have different stiffness.

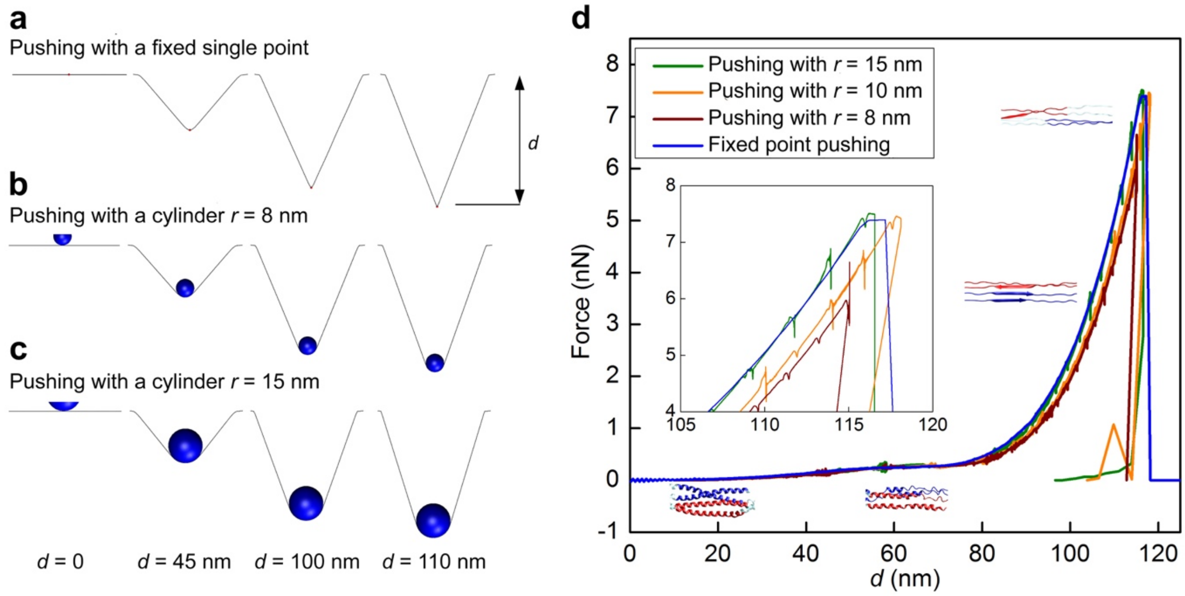

Supplementary Fig. 6. Lamin filament mechanics is not sensitive to cantilever tip curvature. (**a – c**) Simulation analysis showed that pushing with spheres of finite radii (AFM tip) is equivalent to pushing with a single fixed point. The AFM cantilever tip is represented by a sphere of finite radius (*r*). The tip was pushed in the middle of a single lamin filament of 100 nm length and with two fixed ends. The deformation snapshots at different displacement and the full force-displacement curves are given for the tips with different radii. For the pushing test with a fixed single point, only the middle of filament was subjected to the pushing force. (**d**) The different tests provided very similar results for the FE curves demonstrating that the loading can be simplified without modeling the contact between the cantilever tip and the filament. The structural states during the pushing of a lamin filament are shown at corresponding positions along the FE curves.

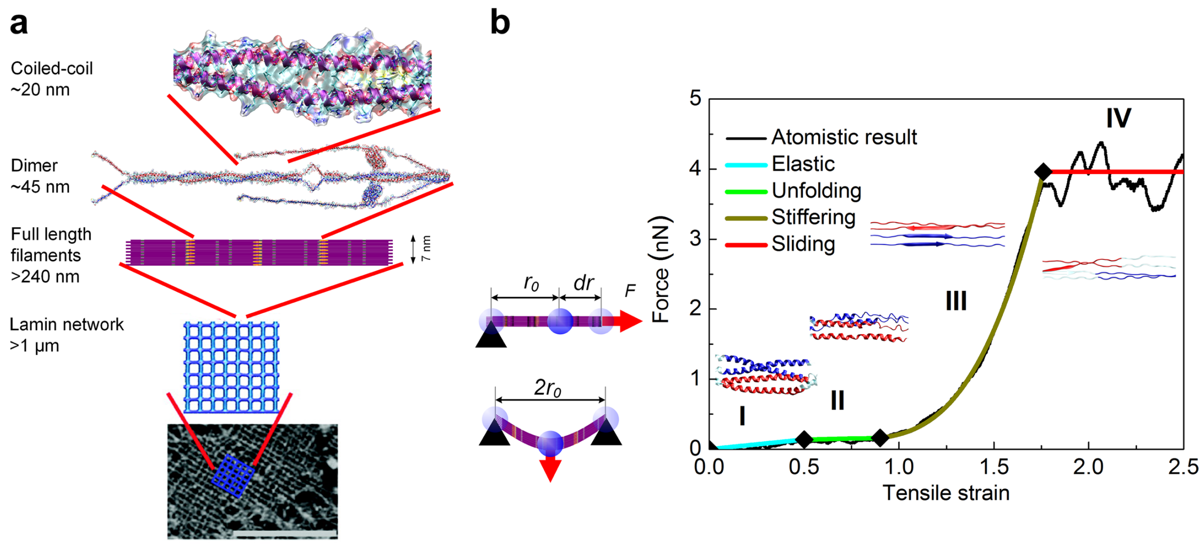

Supplementary Fig. 7. Stretching a lamin filament in the meshwork leads to similar structural transitions as pushing a filament. (**a**) Cartoon depicting the scale of meshwork to filaments to a dimer to an α-helical coiled-coil. Scale bar in the nuclear membrane image is 1 μm. (**b**) MD simulations of stretching a lamin filament along the horizontal plane of the lamin meshwork show (I) elastic stretching, (II) unfolding of α-helical coiled-coils, (III) stiffening because of α-helix to β-sheet transition, and (IV) sliding of the β-sheets leading to filament failure. Except the sliding of β-sheets at failure, the same structural states were observed when a lamin filament was pushed in the meshwork (**Supplementary Fig. 6**). Figure adapted from ref. [1].

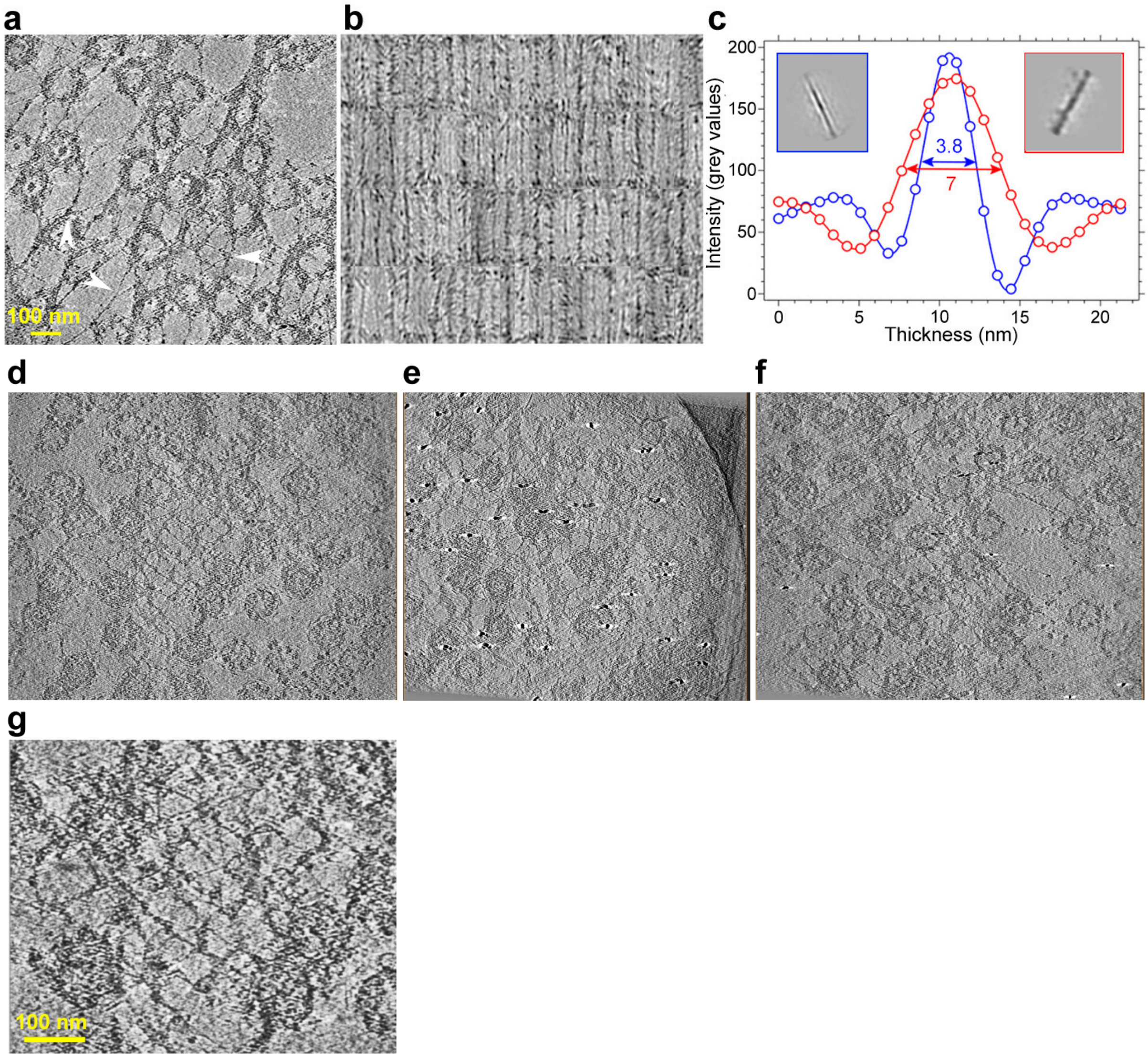

Supplementary Fig. 8. Lamin filaments analyzed by cryo-ET. (**a**) A 10 nm slice through a tomogram of spread NE showing the nuclear lamina of *X. laevis* oocyte. Lamin filaments often interact laterally to form thicker filaments (arrowheads). The filamentous lamin meshwork is anchored at NPCs (round densities). (**b**) A gallery of single lamin filaments (n = 450) extracted *in silico* from the tomograms. (**c**) Density lines through the rod-like structure of two structural class-averages (framed blue and red) indicating filament thickness of ≈3.8 nm and ≈7 nm in agreement with a tetramer and lateral association of tetramers, respectively. The filament dimensions are in agreement with previously measured nuclear lamin filaments in the MEF nuclei[7]. The structural class averages (the two prominent classes are shown in **c**, insets) were calculated as reported in ref. *7*. In brief, the filaments were identified and 1400 sub-volumes of 64 x 64 x 64 pixels (pixel size 1.36 nm, binning 2) were extracted. The filaments in these sub-volumes were then aligned to a cylindrical template parallel to the *y*-axis and projected in the *z*-direction. These projection images were then visually inspected with an interactive tool (available upon request) in MATLAB (Mathworks, Natick, USA) and subjected to 2D classification using Relion[8]. (**d – f**) More examples of 10 nm slices through tomograms of spread NE showing the nuclear lamina of *X.*

laevis oocyte. A total of 13 tilt-series were acquired. Fiducial gold nanoparticles are seen in (**e**). (**g**) Zoom of the slice shown in (**d**). Scale bar shown in (**a**) is the same for (**d – f**).

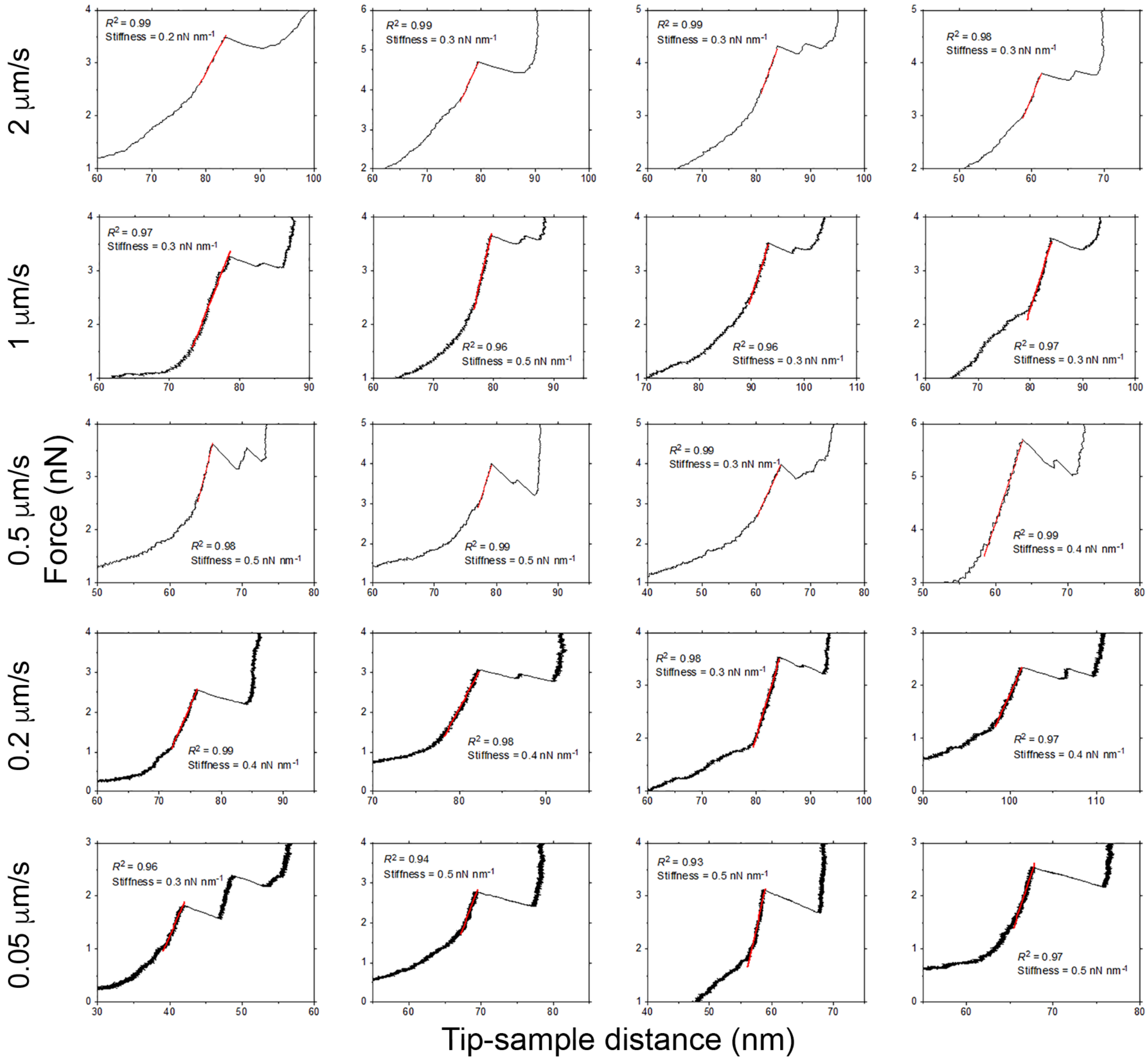

Supplementary Fig. 9. Linear fits of FE curve regions used to determine the filament stiffness. Representative individual FE curves (magnified view) showing the mechanical behavior of lamin filaments under force at different pushing speeds. The linear fits (red lines) used to determine the stiffness of the filament before failure are superpositioned on the original curves. R^2 values were >0.93 with most close to 0.99. The curves shown were chosen at random. Total number of stiffness curves analysed, $n = 1946$.

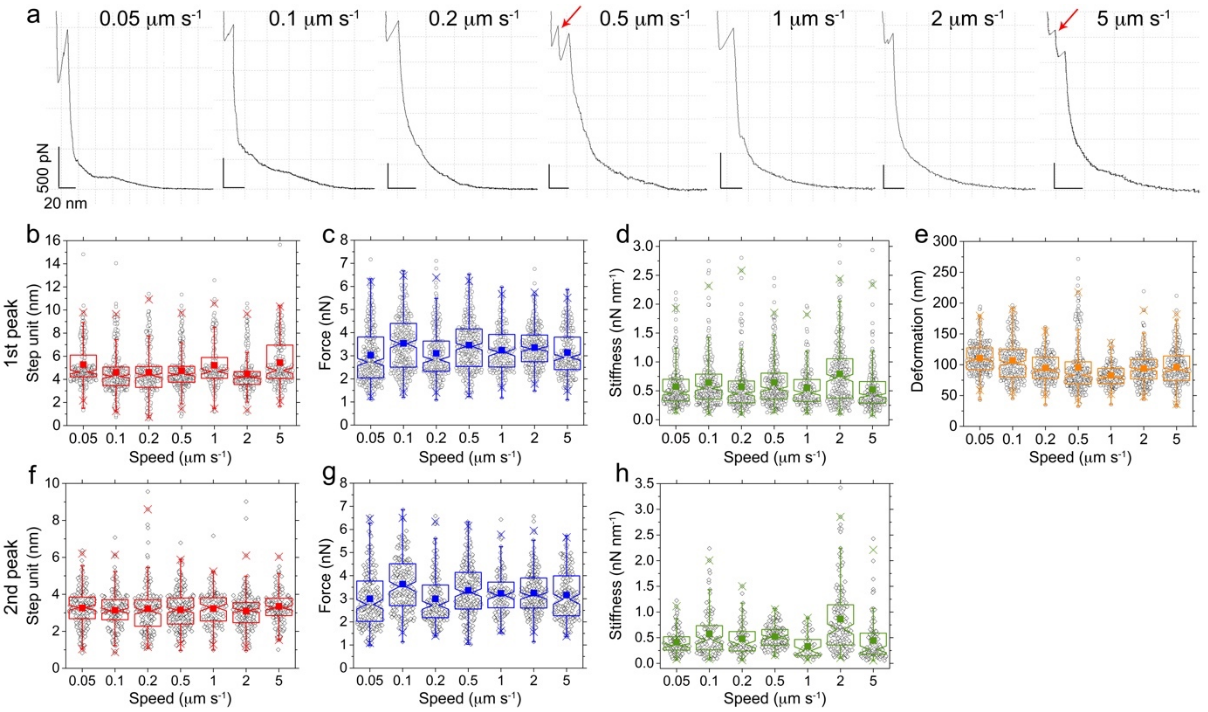

Supplementary Fig. 10. Notch box-plots of the mechanical parameters at increasing loading rates. (**a**) Representative FE curves showing the mechanical behavior of lamin filaments under force. In some cases, after the first failure peak a second peak (red arrows) was observed. The FE curves were independent of the loading rates suggesting a similar load-bearing mechanism of lamin filaments at different loading rates. (**b – e**) The step unit, force, stiffness and deformation of the first peak (n =1946 events), and (**f – h**) the second peak (n =1221 events for step unit and force each, and n = 692 events for stiffness) did not change with the pushing speed. Each notch-box includes 25 – 75% of the data, and each black circle denotes a single force peak event from a filament. The solid squares denote the mean and the whiskers signify 1 – 99% of the data. The number of events analyzed at each speed for both the peaks is reported in **Supplementary Table 2**.

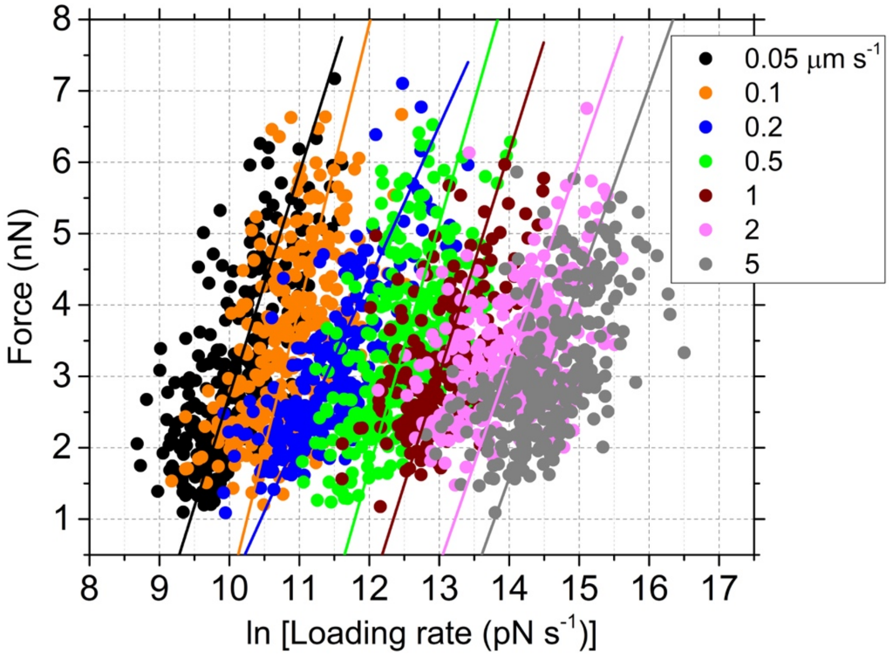

Supplementary Fig. 11. Apparent failure of lamin filaments is independent of loading rate. Each solid circle denotes a single force peak event detected in an FE curve. The number of events (*n*) at 0.05 μm s^{-1}, *n* = 321; 0.1 μm s^{-1}, *n* = 301; 0.2 μm s^{-1}, *n* = 239; 0.5 μm s^{-1}, *n* = 313; 1 μm s^{-1}, *n* = 203; 2 μm s^{-1}, *n* = 293; 5 μm s^{-1}, *n* = 276. The failure forces of lamin were independent of the loading rate (stiffness × speed).

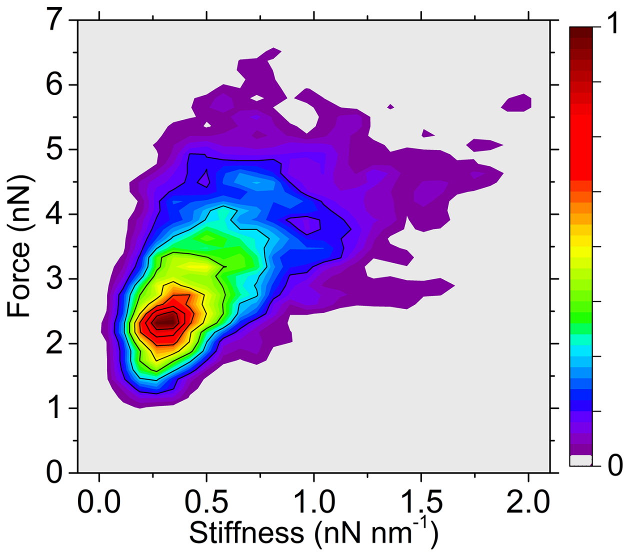

Supplementary Fig. 12. Correlation between force and stiffness of lamin filaments. Density map showing that the stiffer a filament becomes under force, higher the forces it can tolerate before failure. Color scale shows the normalized density.

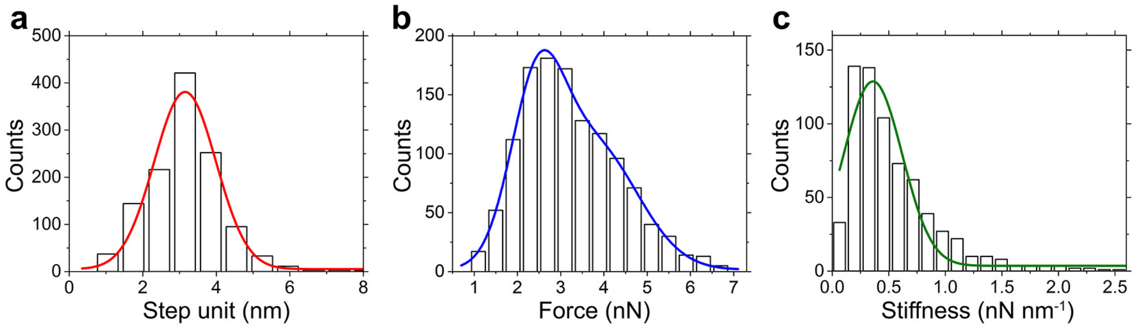

Supplementary Fig. 13. Analysis of the second peak in the FE curves of lamin filaments. Data at different loading rates was pooled to plot histograms of (**a**) step unit (3.2 ± 0.9 nm, ± S.D., $n = 1221$), (**b**) failure force (2.5 ± 0.6 nN, 3.7 ± 1.0 nN, $n = 1221$), and (**c**) stiffness (0.36 ± 0.26 nN nm^{-1}, $n = 692$), which were all similar to the values obtained for the first peak (**Fig. 2**).

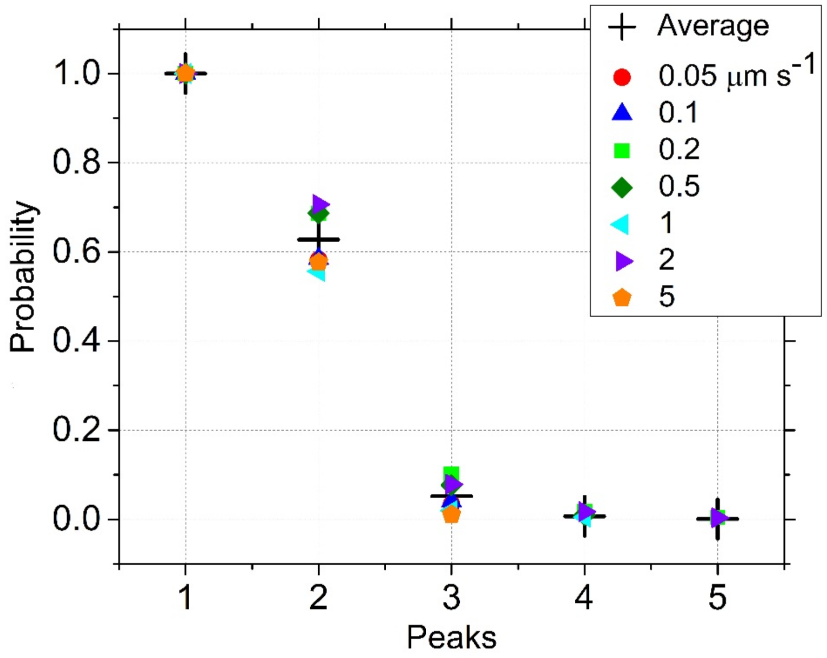

Supplementary Fig. 14. The detection probability of the mechanical failure of lamin filaments. The probabilities (normalized with respect to the first failure peak) measured in the FE curves of lamin filaments. Because the third, fourth and fifth peaks appeared rarely, only the first two peaks were considered for determining the step unit, force, stiffness and deformation. The values are reported in **Supplementary Table 3**.

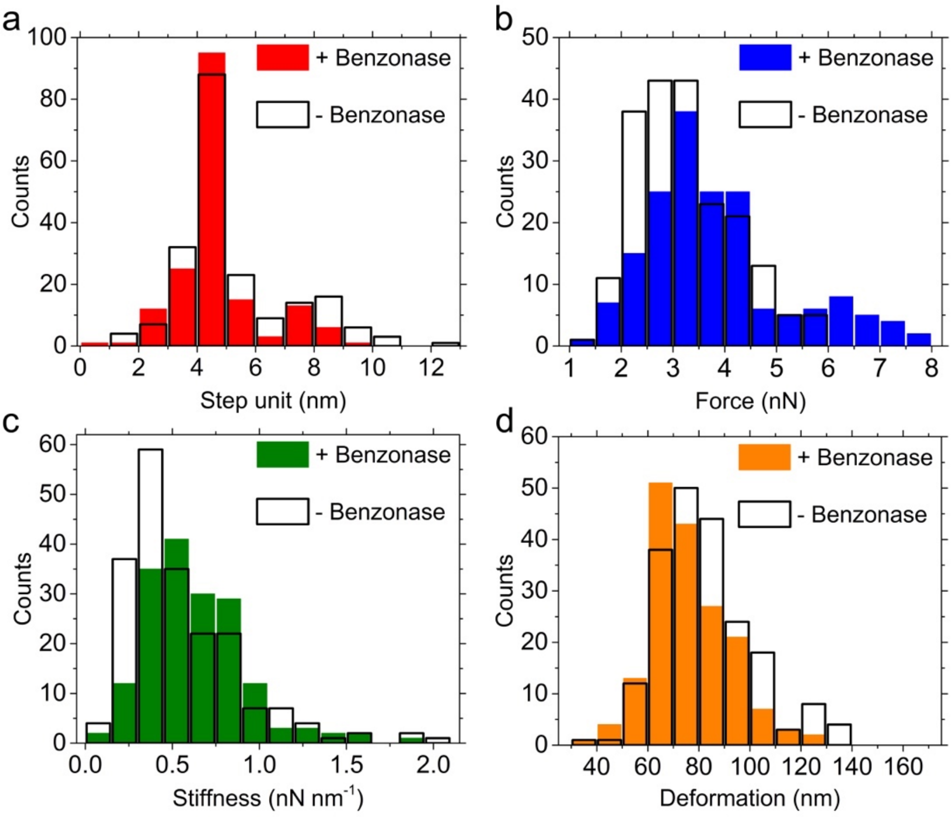

Supplementary Fig. 15. Benzonase nuclease treatment does not change lamin mechanics. (**a**) Step unit, (**b**) force, (**c**) stiffness and (**d**) deformation of lamin filaments in the meshwork of *X. laevis* nuclear envelopes were measured after nuclease treatment at a pushing speed of 1 μm s^{-1}. The values of the parameters were comparable to those obtained without nuclease treatment measured at 1 μm s^{-1}, suggesting that chromatin, ribonucleoproteins or RNA were not present in the experimental system and did not influence the mechanical properties of lamins in our experiments. – Benzonase-free NEs, n = 204 for each parameter; + Benzonase-treated NEs, n = 172 for each parameter. The mean ± S.D. of different parameters are: stiffness – 0.55 ± 0.33 nN nm^{-1} (without benzonase) and 0.63 ± 0.29 nN nm^{-1} (benzonase); failure force – 3.3 ± 1.0 nN (without benzonase) and 3.8 ± 1.4 nN (benzonase); deformation – 83 ± 19 nm (without benzonase) and 76 ± 15 nm (benzonase); step unit – 5.2 ± 1.9 nm (without benzonase) and 4.7 ± 1.4 (benzonase).

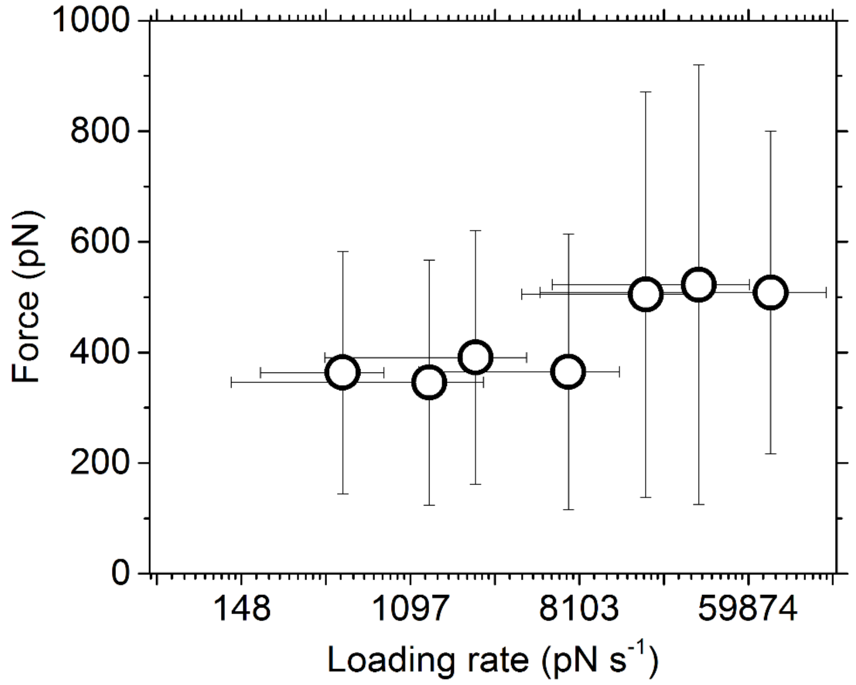

Supplementary Fig. 16. Mechanical response of lamin filaments at low force is independent of the loading rate. Similar to the failure force of the filament after stiffening, the force at which the plateau was observed in the low force regime did not change with increasing loading rates. The data points denote the average values ± standard deviations of the forces (vertical error bars) and loading rates (horizontal error bars). The number of events (n) at 0.05 μm s^{-1}, n = 112; 0.1 μm s^{-1}, n = 57; 0.2 μm s^{-1}, n = 77; 0.5 μm s^{-1}, n = 56; 1 μm s^{-1}, n = 30; 2 μm s^{-1}, n = 47; 5 μm s^{-1}, n = 53.

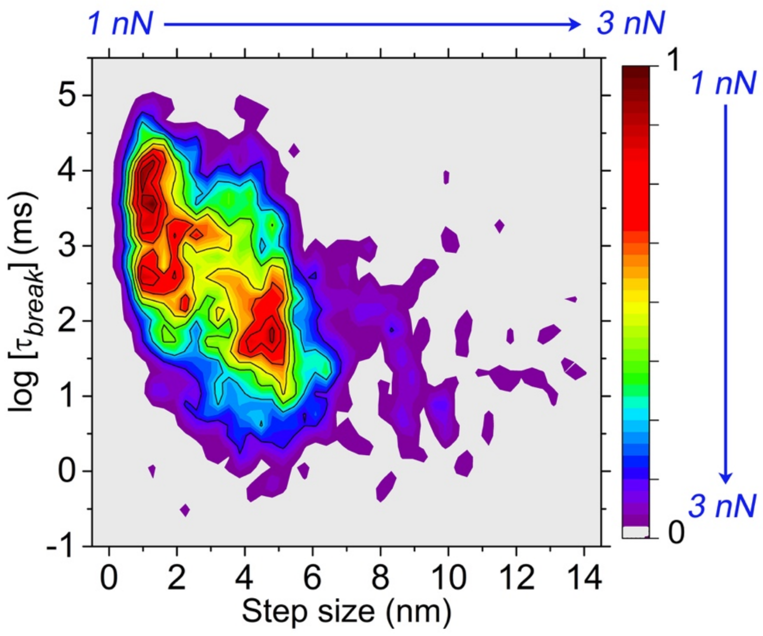

Supplementary Fig. 17. Lifetime of lamin filaments at constant loads. Density map showing the exponential decrease of the lifetime, τ_{break}, with the step size (assigned to α-helix coiled-coil and β-sheet). Two dense populations (red areas) can be clearly seen in the plot. The axes (top and right) denote the trend of F_{load}. α-helical elements (1 – 2 nm) can bear forces up to 1 nN for up to a hundred milliseconds, whereas tetramers (4 nm) stiffen at high forces (3 nN) and fail within a few milliseconds. Color scale denotes the normalized density.

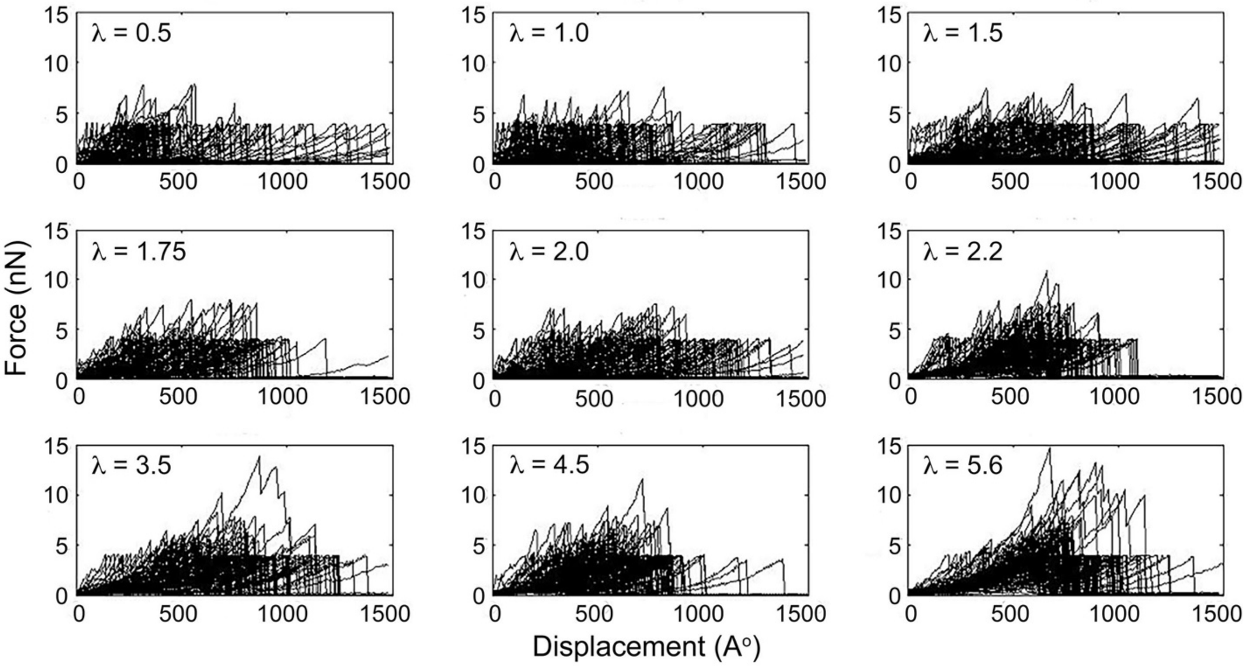

Supplementary Fig. 18. FE curves from MD simulations of pushing single lamin filaments in meshworks of increasing power values ($\lambda = 0.5 - 5.6$). Lamin filaments were pushed *in silico* with a constant velocity (0.1 mm s^{-1}). Meshwork of each λ value was generated randomly, and thereafter tested independently for generating the FE curves 100 individual times. All the FE curves for each λ value are shown in each panel ($\lambda = 0.5$, 1.0, 1.5, 1.75, 2.0, 2.2, 3.5, 4.5 and 5.6).

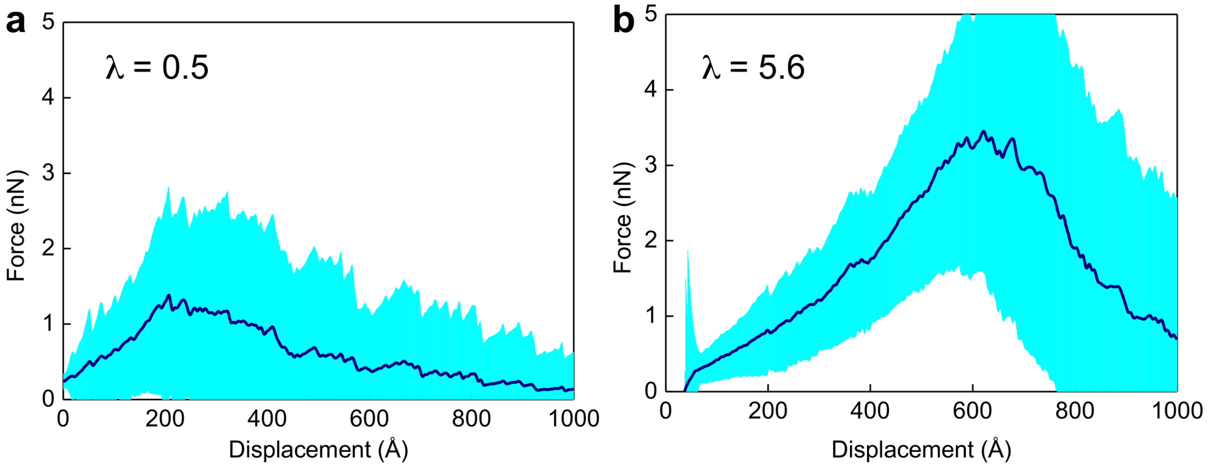

Supplementary Fig. 19. Average force and displacement of lamin filaments depends on meshwork connectivity. (**a, b**) Superimposition of simulated FE curves (turquoise) from meshworks with $\lambda = 0.5$ and 5.6, respectively (n = 100 independent simulations on each network). The average curves (dark blue) clearly show that the filaments can withstand higher forces and deform more in the connected meshwork ($\lambda = 5.6$).

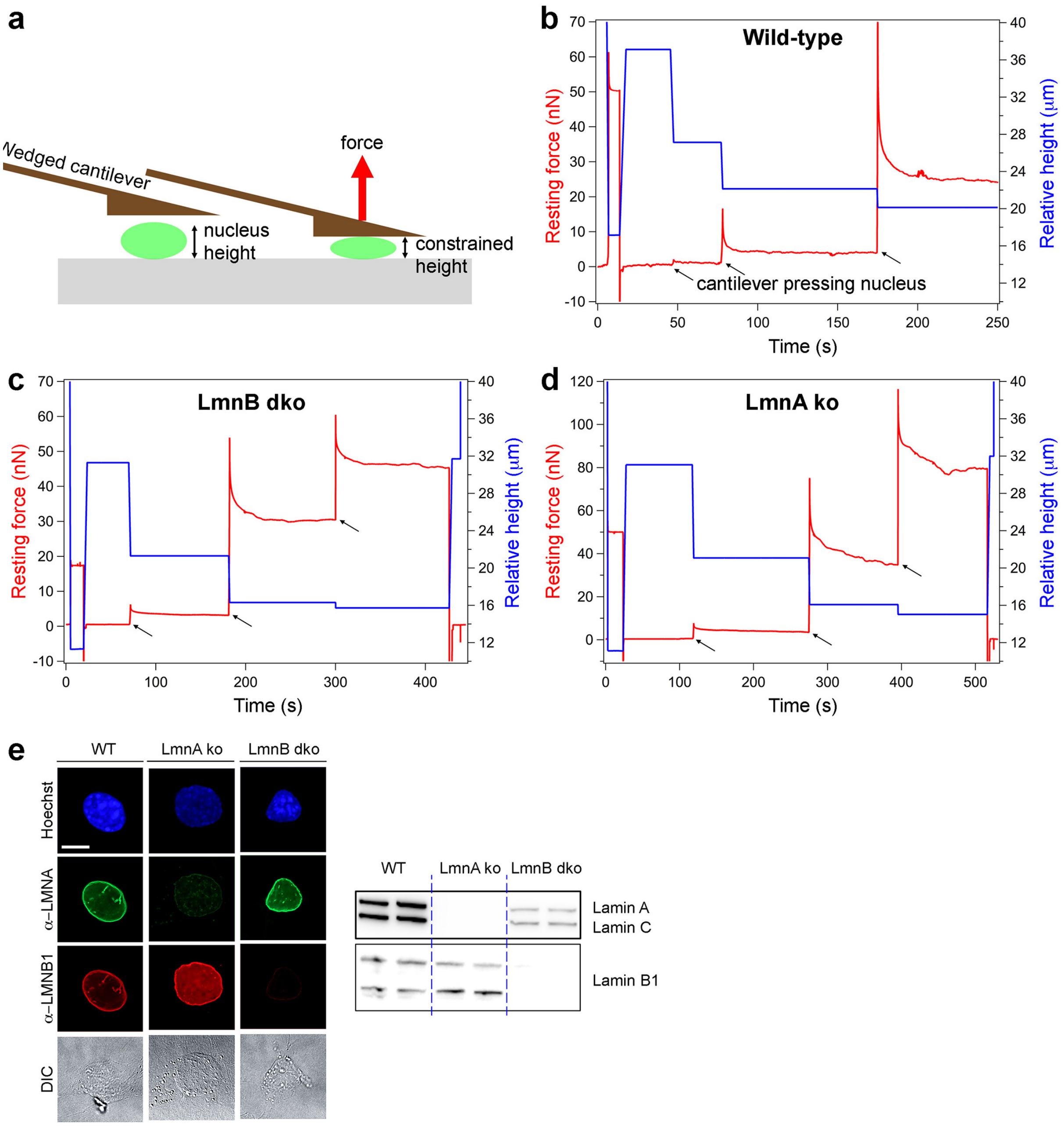

Supplementary Fig. 20. Force resistance of isolated MEF nuclei. (**a**) A single isolated nucleus was subjected to a compression force by confining it between two parallel plates, *i.e.*, between a flat-wedged cantilever and the glass surface. By doing so, the distance between the cantilever and the surface was adjusted such that the nucleus was compressed to 10 μm, 5 μm and 3 μm from its initial height (blue trace) (see **Methods**). (**b**) Nuclei were isolated from wild-type MEF, (**c**) lamin B1/B2 double knock-out (LmnB dko), (**d**) lamin A knock-out (LmnA ko), constrained to specific heights by the cantilever, and the resting force measured (force reaching steady state after the initial rise, shown by black arrows) (**Supplementary Fig. 21**). The resting force is the resistance (counterforce) of a nucleus to compression force and may be considered as an indicator of nucleus stiffening. A stiffer nucleus will be able to resist the compressing force more than a softer nucleus. Shown are representative traces from each nucleus type. WT nuclei, $n = 16$; LmnB dko nuclei, $n = 14$; LmnA ko nuclei,

n = 10. (**e**) Immunofluorescence images (N = 4, N is the independent experiments for each nucleus type; n = 25, n is the number of nuclei of each type) and Western blot analysis showing the knockout of LMNA (ko) and LMNB (dko) genes. Scale bar, 10 μm.

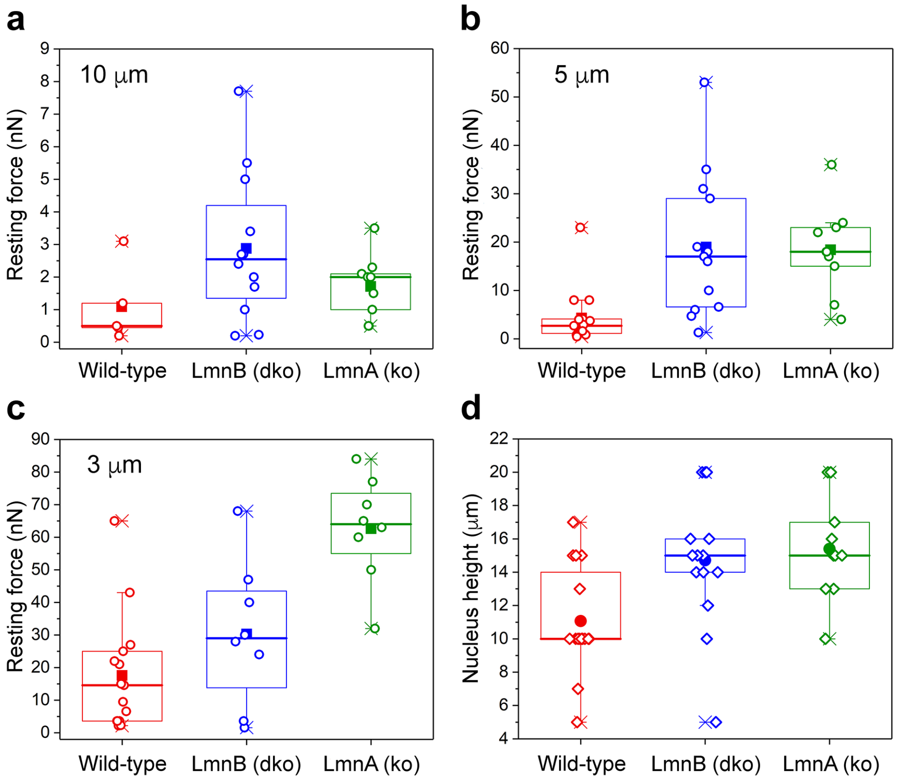

Supplementary Fig. 21. Force resistance of isolated MEF nuclei. Data of resting force experiments of MEF nuclei expressing lamins A/C and B1/B2 (wild-type), only A-type lamins A/C (Lmn B dko) or only B-type lamins B1/B2 (Lmn A ko) are plotted. (**a, b**) MEF nuclei expressing only A-type or B-type lamins showed higher resting force or increased stiffness compared to wild-type nuclei when compressed to 10 μm and 5 μm heights using flat-wedged cantilevers. (**c**) Upon increasing the compression to 3 μm, lamin B expressing nuclei showed the highest stiffness indicated by an increase in the resistance force by ~200% compared to wild-type nucleus. (**d**) Nuclei heights measured before applying compression forces with a flat-wedged cantilever. WT nuclei, $n = 16$; LmnB dko nuclei, $n = 14$; LmnA ko nuclei, $n = 10$. Each box includes 25 – 75% of the data, and each circle denotes a single nucleus. The solid squares denote the mean values, the bold horizontal lines inside the box denote the median values, and the whiskers signify 1 – 99% of the data.

Supplementary Table 1. Relative populations of the plateau and the intermediate peak (low force regime) in the FE curves of lamin filaments.

**Speed** ($\mu m\ s^{-1}$)	**Plateau** (*n*, events)	**Intermediate peak** (*n*)	**Total FE curves** (*N*)	**Plateau** (%)	**Intermediate peak** (%)
0.05	112	15	321	34.9	4.7
0.1	57	21	301	18.9	7.0
0.2	77	38	239	32.2	15.9
0.5	56	43	313	17.9	13.7
1	30	9	203	14.8	4.4
2	47	39	293	16.0	13.3
5	53	10	276	19.2	3.6

Supplementary Table 2. Statistics of the two peaks from constant velocity force-extension experiments of lamin filaments.

**Peak**	**Number of events** (*n*)	**Speed** (μm s^{-1})
1 (all parameters)	321	0.05
	301	0.1
	239	0.2
	313	0.5
	203	1
	293	2
	276	5
2 (step unit and force)	187	0.05
	176	0.1
	164	0.2
	215	0.5
	113	1
	207	2
	159	5
2 (stiffness)	106	0.05
	104	0.1
	74	0.2
	121	0.5
	43	1
	144	2
	100	5

Supplementary Table 3. Relative population of the peaks observed in FE curves in constant velocity experiments.

**Speed** (μm s^{-1})	**1st peak % ± error** (n =)	**2nd peak**	**3rd peak**	**4th peak**	**5th peak**
0.05	1 (321)	0.6 ± 0.03 (187)	0.03 ± 0.01 (11)	-	-
0.1	1 (301)	0.6 ± 0.03 (176)	0.04 ± 0.01 (12)	-	-
0.2	1 (239)	0.7 ± 0.03 (164)	0.1 ± 0.02 (24)	0.02 ± 0.01 (4)	0.004 ± 0.004 (1)
0.5	1 (313)	0.7 ± 0.03 (215)	0.08 ± 0.02 (24)	0.01 ± 0.006 (3)	-
1	1 (203)	0.6 ± 0.03 (113)	0.02 ± 0.01 (4)	0.005 ± 0.005 (1)	-
2	1 (293)	0.7 ± 0.03 (207)	0.08 ± 0.02 (23)	0.02 ± 0.008 (5)	0.003 ± 0.003 (1)
5	1 (276)	0.6 ± 0.03 (159)	0.01 ± 0.006 (3)	-	-
Total	1 (1946)	0.6 ± 0.01 (1221)	0.05 ± 0.005 (101)	0.007 ± 0.002 (13)	0.001 ± 0.0007 (2)

References

1 Qin, Z. & Buehler, M. J. Flaw tolerance of nuclear intermediate filament lamina under extreme mechanical deformation. *ACS Nano* **5**, 3034-3042 (2011).

2 Hanson, L. *et al.* Vertical nanopillars for in situ probing of nuclear mechanics in adherent cells. *Nat Nanotechnol* **10**, 554-562 (2015).

3 Qin, Z., Kreplak, L. & Buehler, M. J. Nanomechanical properties of vimentin intermediate filament dimers. *Nanotechnology* **20**, 425101 (2009).

4 Shimi, T. *et al.* Structural organization of nuclear lamins A, C, B1, and B2 revealed by superresolution microscopy. *Mol Biol Cell* **26**, 4075-4086 (2015).

5 Aebi, U., Cohn, J., Buhle, L. & Gerace, L. The nuclear lamina is a meshwork of intermediate-type filaments. *Nature* **323**, 560-564 (1986).

6 Eibauer, M. *et al.* Structure and gating of the nuclear pore complex. *Nat Commun* **6** (2015).

7 Turgay, Y. *et al.* The molecular architecture of lamins in somatic cells. *Nature* **543**, 261-264 (2017).

8 He, S. & Scheres, S. H. W. Helical reconstruction in RELION. *J Struct Biol* **198**, 163-176 (2017).
