# Supplementary material for: Nonlinear mechanics of lamin filaments and the meshwork topology build an emergent nuclear lamina
Source: Nat Commun. 2020 Dec 4;11:6205. doi: 10.1038/s41467-020-20049-8 (PMC7718915; doi:10.1038/s41467-020-20049-8)
Supplement: Supplementary file 3 — Description of Additional Supplementary Files [file 41467_2020_20049_MOESM3_ESM.pdf]

Description of Additional Supplementary Files

File Name: Supplementary Movie 1
Description: Molecular dynamics simulations of pushing lamin filaments from a meshwork of λ = 0.5. Analysis presented in **Fig. 6**.

File Name: Supplementary Movie 2
Description: Molecular dynamics simulations of pushing lamin filaments from a meshwork of λ = 5.6. Analysis presented in **Fig. 6**.
